# Supplementary material for: Milk Consumption Decreases Risk for Breast Cancer in Korean Women under 50 Years of Age: Results from the Health Examinees Study
Source: Nutrients. 2019 Dec 21;12(1):32. doi: 10.3390/nu12010032 (PMC7019619; doi:10.3390/nu12010032)
Supplement: Supplementary file 1 [file nutrients-12-00032-s001.pdf]

Table S1. Hazard ratios (HRs) and 95% confidence intervals (CIs) of breast cancer according to yogurt consumption.

	Yogurt Consumption (Serving)				
	**<1/week**	**1/week**	**2-6/week**	**≥1/day**	***p* for Trend [b]**
**Age <50 years**					
Person-years	100,729	39,212	29,867	23,229	
Number	15,624	6134	4500	3545	
Breast Cancer Cases	74	28	29	19	
HR (95% CI) [a]	1.00 (ref)	0.94 (0.61, 1.46)	1.27 (0.82, 1.97)	1.03 (0.61, 1.74)	0.6636
**Age ≥50 years**					
Person-years	161,894	51,439	42,369	45,751	
Number	26,042	8419	6808	7248	
Breast Cancer Cases	112	27	41	29	
HR (95% CI) [a]	1.00 (ref)	0.73 (0.48, 1.11)	1.29 (0.90, 1.86)	0.82 (0.54, 1.25)	0.7101

[a] HR (95% CI) : adjusted for BMI (kg/m^2, continuous), total energy intake (kcal/day, continuous), educational level (middle school or less, high school or college, undergraduate or more), parity (nulliparous, 1, 2, ≥3), age at first birth (nulliparous, aged <25 years at first birth, aged ≥25 at first birth), age at menarche (<15, 15, ≥16 years), oral contraceptive use (never, ever), regular exercise (no, yes), alcohol consumption (never, ever), and the presence of a family history of breast cancer (no, yes); [b] P for trend was calculated using the median value of each category as a continuous variable.

Table S2. Hazard ratios (HRs) and 95% confidence intervals (CIs) of breast cancer according to cheese consumption.

	Cheese Consumption (Serving)			
	**<1/week**	**1/week**	**≥2/week**	***p* for Trend [b]**
**Age <50 years**				
Person-years	173,973	11,894	7171	
Number	26,666	1974	1163	
Breast Cancer Cases	128	14	8	
HR (95% CI) [a]	1.00 (ref)	1.57 (0.89, 2.75)	1.43 (0.69, 2.96)	0.2461
**Age ≥50 years**				
Person-years	274,297	13,592	13,565	
Number	43,913	2308	2296	
Breast Cancer Cases	183	9	17	
HR (95% CI) [a]	1.00 (ref)	0.87 (0.44, 1.71)	1.61 (0.97, 2.68)	0.0757

[a] HR (95% CI) : adjusted for BMI (kg/m^2, continuous), total energy intake (kcal/day, continuous), educational level (middle school or less, high school or college, undergraduate or more), parity (nulliparous, 1, 2, ≥3), age at first birth (nulliparous, aged <25 years at first birth, aged ≥25 at first birth), age at menarche (<15, 15, ≥16 years), oral contraceptive use (never, ever), regular exercise (no, yes), alcohol consumption (never, ever), and the presence of a family history of breast cancer (no, yes); [b] P for trend was calculated using the median value of each category as a continuous variable.
